# SemClinBr - a multi-institutional and multi-specialty semantically annotated corpus for Portuguese clinical NLP tasks

**DOI:** 10.1186/s13326-022-00269-1

**Published:** 2022-05-08

**Authors:** Lucas Emanuel Silva e Oliveira, Ana Carolina Peters, Adalniza Moura Pucca da Silva, Caroline Pilatti Gebeluca, Yohan Bonescki Gumiel, Lilian Mie Mukai Cintho, Deborah Ribeiro Carvalho, Sadid Al Hasan, Claudia Maria Cabral Moro

**Affiliations:** 1 Health Technology Program, Pontifical Catholic University of Paraná, Rua Imaculada Conceição, 1155 - Curitiba, Paraná, 80215-901 Brazil; 2 AI Lab, Philips Research North America, Cambridge, MA USA

**Keywords:** Natural language processing, Semantic annotation, Clinical narratives, Corpora, Gold standard

## Abstract

**Background:**

The high volume of research focusing on extracting patient information from electronic health records (EHRs) has led to an increase in the demand for annotated corpora, which are a precious resource for both the development and evaluation of natural language processing (NLP) algorithms. The absence of a multipurpose clinical corpus outside the scope of the English language, especially in Brazilian Portuguese, is glaring and severely impacts scientific progress in the biomedical NLP field.

**Methods:**

In this study, a semantically annotated corpus was developed using clinical text from multiple medical specialties, document types, and institutions. In addition, we present, (1) a survey listing common aspects, differences, and lessons learned from previous research, (2) a fine-grained annotation schema that can be replicated to guide other annotation initiatives, (3) a web-based annotation tool focusing on an annotation suggestion feature, and (4) both intrinsic and extrinsic evaluation of the annotations.

**Results:**

This study resulted in SemClinBr, a corpus that has 1000 clinical notes, labeled with 65,117 entities and 11,263 relations. In addition, both negation cues and medical abbreviation dictionaries were generated from the annotations. The average annotator agreement score varied from 0.71 (applying strict match) to 0.92 (considering a relaxed match) while accepting partial overlaps and hierarchically related semantic types. The extrinsic evaluation, when applying the corpus to two downstream NLP tasks, demonstrated the reliability and usefulness of annotations, with the systems achieving results that were consistent with the agreement scores.

**Conclusion:**

The SemClinBr corpus and other resources produced in this work can support clinical NLP studies, providing a common development and evaluation resource for the research community, boosting the utilization of EHRs in both clinical practice and biomedical research. To the best of our knowledge, SemClinBr is the first available Portuguese clinical corpus.

## Background

In the past two decades, natural language processing researchers developed a large amount of work focusing on extracting and identifying information from unstructured data (i.e., clinical narratives) stored in electronic health records [1, 2]. In addition, the scientific community has had an increasing demand for corpora with high-quality annotations to develop and validate their methods [3]. Semantically annotated corpora can be very useful for both the development and evaluation of NLP and machine learning (ML) algorithms aimed at mining information from EHRs to better utilize EHR data in clinical practice and improve the availability of resources for biomedical research [4]. In the clinical domain, this could be a major issue, as privacy restrictions applied to EHR data do not allow personal health information (PHI) to be openly shared for research. Therefore, it is mandatory to de-identify (anonymize) the patient personal data before use, as determined by the Health Insurance Portability and Accountability Act (HIPAA) [5].

It is difficult to find a unique corpus that can be potentially applied to several clinical NLP tasks. Such a corpus would be an annotated collection with broad scope and in-depth characteristics, while at the same time, presenting entities with high granularity accompanied by comprehensive documents from the perspective of clinical specialties, annotation type, and institutional origin. Working with a language outside the scope of English could be another barrier, as most annotations are in English, including studies developed for shared tasks and challenges [6–13]. Very few initiatives have shared clinical reference corpora in other languages [14, 15], and to the best of our knowledge, none of them are in Brazilian Portuguese (pt-br).

With the objective of structuring a background to support the biomedical NLP field for pt-br and address the gaps in broad clinical corpora outside the English scope, a semantically annotated corpus was developed in this study, to assist clinical NLP tasks, both for the evaluation and development of these tasks, using real clinical text from multiple institutions, medical specialties, and document types. An annotation schema was defined with fine granular entities, and annotation guidelines were provided as reference for the annotators. A new annotation tool was further developed with features to enable faster and more reliable annotation. The main contributions can be highlighted as follows: (1) SemClinBR, is the first semantically annotated clinical corpus in pt-br; (2) Our clinical corpora survey lists common steps and lessons learned in corpus development; (3) A replicable annotation schema is provided; and (4) A web-based annotation tool with an annotation assistant is incorporated.

### Annotation initiatives

The use of statistical NLP and ML allows researchers to automatically retrieve information from biomedical texts and increases the need for gold standard (or ground-truth) corpora to support supervised strategies. Because of the cost and issues related to annotation projects [16], the scientific community must share efforts to boost the use of biomedical data to provide researchers with a common evaluation and development environment, which could make it easier to benchmark different methods.

The prevalence of biomedical literature corpora over clinical corpora is evident in recent studies [6, 16, 17]. While the first (usually) deals with open scientific information (e.g., scientific papers and gene data), the second utilizes EHR personal data, which require, among others, anonymization and ethical committee approval prior to releasing the information to the research community. Nevertheless, various clinical semantic annotation initiatives have been developed and shared over the last ten years. An overview of some of these studies is provided in this section, listing some of the common features to better characterize them. The focus is on studies working with categorical labeling (i.e., applying categories / types to the concepts in the text), while ignoring studies performing term-to-concept annotation [18], which link the concepts in the text to corresponding entries in certain terminologies (e.g., UMLS – Unified Medical Language System [19]).

Shared tasks and research challenges are well-known sources of clinically annotated data, as they focus on the development of a specific trending clinical NLP task, to provide a common evaluation background for the scientific community. The i2b2 challenges have covered important clinical NLP tasks over almost a decade, including clinical data de-identification [20, 21], patient smoking status detection [22], obesity and co-morbidities recognition [23], medication extraction [24], concepts / assertions / relation extraction [8], co-reference resolution [25], temporal relation extraction [26], and heart disease risk factor identification [27]. Some corpora annotations are described in specific papers [13, 28–30] and are available to the research community on the i2b2 webpage. Another important initiative is the SemEval evaluation series, which focus on general semantic analysis systems, not limited to the biomedical / clinical domain; however, they already share corpora for specific clinical tasks, such as the “Analysis of Clinical Text” task on SemEval-2014 [11], SemEval-2015 [12], the “Clinical TempEval” task in SemEval-2016 [31], and SemEval-2017 [32]. The ShARe / CLEF eHealth labs shared a set of annotated clinical notes for two shared-task editions [9, 33] and three different NLP tasks consisting of: (1) named entity recognition (NER) and normalization of disorders, (2) normalization of acronyms and abbreviations, and (3) patient information retrieval.

To develop and evaluate the environment for clinical information extraction systems, the CLinical E-Science Framework (CLEF) project built a semantically annotated corpus of clinical text [6]. The project labeled entities, relations, modifiers, co-references, and temporal information within the text using the CLEF project tag set. Because of the large size (20,234 clinical documents), the corpus focused only on patients with neoplasms. In a recent study, Patel et al. [34] built a large clinical entity corpus with 5160 clinical documents from 40 different medical domains. They annotated a set of 11 semantic groups that were mapped to the corresponding UMLS semantic types.

The Temporal Histories of Your Medical Events corpus (THYME) [35] is another example of a gold standard produced by annotating clinical notes. The annotation process focused on event and relation annotations, in particular, temporal information. Finally, the MiPACQ corpus [36] features syntactic and semantic annotations of clinical narratives (127,606 tokens precisely). Semantic labeling followed the UMLS hierarchy of semantic groups [37] to avoid semantic ambiguity.

The discussion so far points to a predominance of corpora built for the English language. There were no studies focusing on clinical semantic annotation of pt-br. However, there is a non-shared corpus in European Portuguese, named the MedAlert Discharge Letters Representation Model (MDLRM), developed by Ferreira et al. [18]. They annotated a set of entities (i.e., condition, anatomical site, evolution, examination, finding, location, therapeutic, date, time, and value) in 90 discharge summaries from a hospital in Portugal, aiming to evaluate an NER task.

Furthermore, there have been some efforts dedicated in other languages, as mentioned below. For Spanish, experts annotated the IxaMed-GS corpus [14] with entities and relations associated with diseases and drugs using an adaptation of the SNOMED-CT tag set. A notable 3-year-long work is the Medical Entity and Relation LIMSI annOtated Text corpus (MERLOT) [15], which produced a corpus of 500 annotated clinical documents for the French language using an entity annotation scheme partially derived from the UMLS semantic groups. The development and analysis of this clinical representation scheme are described in detail in [38].

A recent study focused on German nephrology reports on building a fine-grained annotated corpus following a concept-type organization similar to the UMLS semantic types / groups. The corpus consisted of 118 discharge summaries and 1607 short evolution notes [39]. In addition, for the Swedish language, Skeppstedt et al. [40] annotated a set of highly relevant entities to build a patient overview (disorder, finding, pharmaceutical drugs, and body structure) to train an NER algorithm previously applied to English clinical texts. Their corpus includes 45,482 tokens for the training set and 25,370 tokens for the evaluation set.

To realize cohesive, reliable, unbiased, and fast annotation, most studies share the following common steps.

- double annotation ➔ to reduce bias and improve reliability
- guidelines / scheme definition ➔ to improve reliability and support annotators
- annotation agreement measures ➔ to ensure reliability
- use of an annotation tool ➔ to ease / speed up the annotation work
- annotation characterization (e.g., semantic types, relations) based on the desired task for better scope definition.

Primarily because of annotation costs, issues associated with high generalization and specificity of the annotation, and difficulties in obtaining clinical data, the available corpora do not share all of the following characteristics:

- documents from multiple institutions ➔ different writing and care styles
- multiple types of documents (e.g., discharge summaries and nursing notes) ➔ distinct care phases
- documents from various medical specialties (e.g., cardiology and nephrology), broader clinical views, and care perspectives.
- multiple medical conditions (e.g., diabetes, cardiovascular disease) ➔larger dictionary of terms
- high granularity entity annotation (normally a few entity types are grouped) enables specific labeling
- sharing a detailed annotation guideline ➔ allows replication
- a high number of clinical notes ➔ more representativity and ML training conditions
- scope outside English ➔ boosts research field in other languages

Although there is a lack of large multipurpose corpora, it is also necessary to note the need for a heterogeneous clinical corpus for the scientific community. For example, Deleger et al. [41] argued that most clinical data de-identification systems were tested on corpora composed of a unique or small variety of document types (e.g., discharge summaries and nursing notes) whereas the ideal would be an evaluation using heterogeneous corpora. In addition, when defining the granularity of corpus annotation, one must remember the trade-off between granularity and reliability, as discussed by Crible and Degand [42], prioritizing each of these aspects according to the objectives of the annotation.

Hovy and Lavid [43] referred to corpus annotation as “*adding interpretive information into a collection of texts,*” and described seven main questions in a general annotation project:

1. Representative text selection
2. Concept / theory instantiation (tag set definition + guidelines first draft)
3. Selection of annotators and training (preliminary annotation + guidelines update)
4. Annotation procedure specification (definitive guidelines)
5. Annotation interface design (increase speed and avoid bias)
6. Definition of evaluation measures (satisfactory agreement level – in case of low agreement, return to step 2 – if in good agreement, continue annotation, maintain intermediate checks, improvements, etc.)
7. Finalize annotation and NLP / ML algorithm deployment

Xia and Yetisgen-Yildiz [16] listed the challenges and strategies in clinical corpus annotation and concluded that the medical training of the annotators, in itself, is not enough to ensure that high-quality annotations will be achieved; thus, NLP researchers should be involved in the annotation process as early as possible. Moreover, compared with a typical annotation task, the use of physicians is much more expensive and difficult to schedule. Therefore, depending on the complexity / specialization of the clinical task, annotation by medical students may be a viable alternative.

The reliability of an annotated corpus is another important aspect that should be considered. Most studies rely on inter-annotator agreement (IAA) calculation as the primary metric to assess reliability. Different methods are used to calculate IAA. Artstein and Poesio [44] surveyed most of these methods (e.g., observed agreement, Krippendorff’s alpha, Cohen’s kappa) and discussed their use in multiple annotation tasks. As pointed out by many researchers [14, 41, 45, 46], Cohen’s kappa and other chance correction approaches (which are vastly used in classification tasks) are not the most appropriate measures for named entity annotation because the probability of agreement by chance between annotators is nearly zero when labeling text spans. In addition to the method used to calculate IAA, the question of what measure would represent a high-quality gold standard arises. Hovy and Lavid [43] stated that the annotator manager needs to determine the acceptable IAA values based on the goals when using the corpus to train ML algorithms; one should aim to have sufficient data with realistic agreement values considering the desirable task. In the medical area, a well-known and accepted convention for IAA “strength” values is the one proposed by Landis and Koch [47], in which 0.41 ≤ IAA ≤ 0.60 is moderate, 0.61 ≤ IAA ≤ 0.80 is substantial, and IAA ≥ 0.81 is almost perfect.

Artstein and Poesio [44] claim that an adequate level of agreement for a specific purpose is obscure because different levels of agreement may be good for one purpose and bad for another. They discussed Reidsma and Carletta’s work [48], in which the authors approach the reliability thresholds used in computational linguistics (CL), where IAA ≥ 0.8 is considered to be good and 0.8 > IAA > 0.67 is tolerable. The authors also demonstrated that ML algorithms can tolerate data with low reliability values, and sometimes, a reliability measure of 0.8 is not synonymous of good performance. In other words, agreement metrics are weak predictors of ML performance. This agrees to an extent with the discourse of Roberts et al. [6], who stated that *“The IAAs between double annotators that are given do not therefore provide an upper bound on system performance, but are an indication of how hard a recognition task is.”*

## Methods

**Fig. 1:**
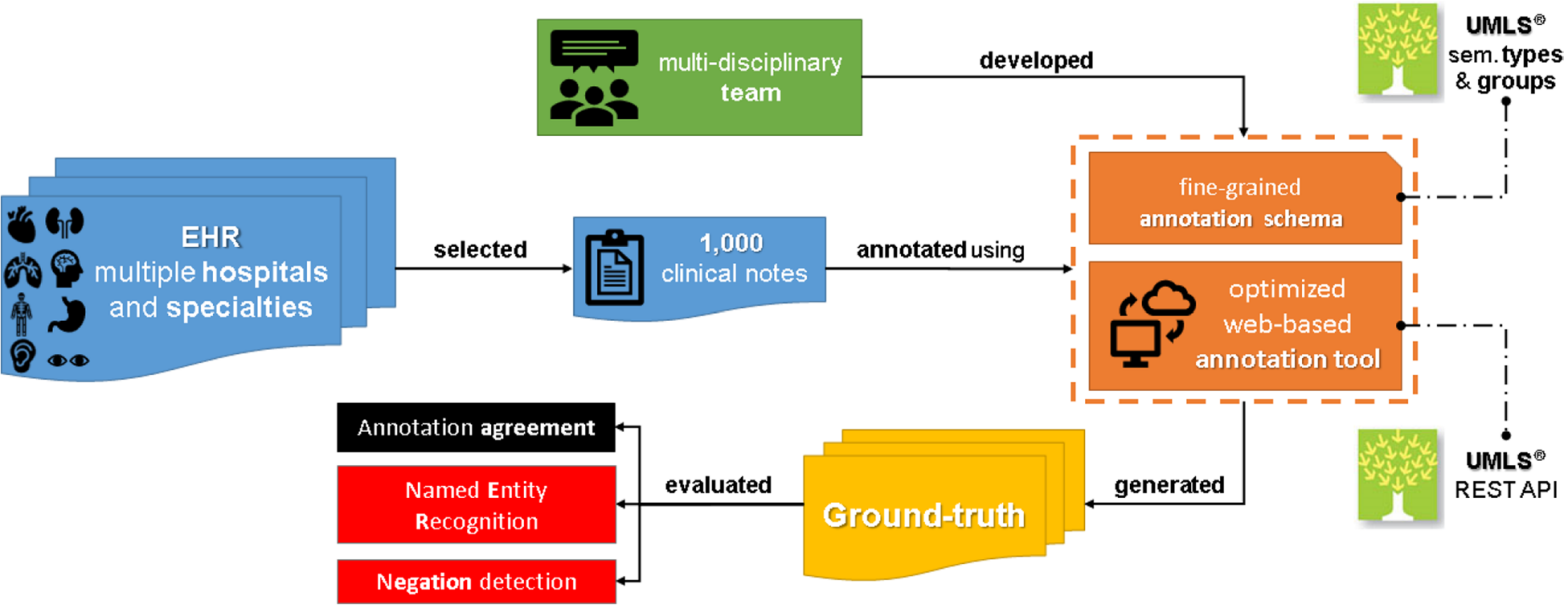
A broad view of SemClinBr corpus development. The diagram is an overview of the SemClinBr corpus development, which shows the selection of thousands of clinical notes from multiple hospitals and medical specialties. A multidisciplinary team developed the elements in orange, representing (i) the fine-grained annotation schema following the UMLS semantic types and (ii) the web-based annotation tool featuring the UMLS REST API. These resources supported the generation of the ground truth (i.e., gold standard), which was evaluated intrinsically (i.e., inter-annotation agreement) and extrinsically in two different NLP tasks (i.e., named entity recognition and negation detection)

In this section, details are provided regarding the EHR data used in our study and all aspects associated with the annotation schema, including the guideline definition, annotation tool development, annotation process itself, corpus reliability calculation, and segmentation of the created ground-truth. A broad perspective on our methodology is presented in Fig. 1.

### Data preparation

The data were obtained from two different sources: (1) a corpus of 2,094,929 entries from a group of hospitals in Brazil generated between 2013 and 2018, and (2) a corpus originating from a university hospital based on entries in the period between 2002 and 2007, which accounts for 5.617 of the entries. In the first dataset, each entry was associated with structured data (e.g., gender, medical specialty, entry date) as well as unstructured data in free-text format, representing sections of a clinical narrative (e.g., disease history, family history, and main complaint). Data were obtained from the records of approximately 553,952 patients. In addition to the multi-institutional aspect of the corpus, it covers various medical specialties (e.g., cardiology, nephrology, and endocrinology) and clinical narrative types (e.g., discharge summaries, nursing notes, admission notes, and ambulatory notes).

The second dataset had only one document type (discharge summaries) and came exclusively from the Cardiology Service Center. The data configuration had structured data (i.e., gender, birth date, start date, end date, and icd-10 code) and only one free-text data field for the discharge summary. The texts from both datasets shared some already known characteristics related to clinical narratives in general [49], such as uncertainty, redundancy (often due to copy and paste), high use of acronyms and medical jargon, misspellings, fragmented sentences, punctuation issues, and incorrect lower- and uppercasing. Some examples of text are presented in Table 1. The first example is a discharge summary from the cardiology section, with the complete history of care provided by regular / long sentences with no apparent format standardization. The second sample shows an ambulatory note describing a patient visit to the nephrology department, which includes concise sentences written in uppercase letters, a high frequency of acronyms, and a lack of punctuation. The third text is a nursing note describing the monitoring of the nursing team by the patient. The de-identification process is described in the “annotation tool” section.

**Table 1:** Samples of different types of clinical narratives from our corpus

Type/Specialty	Original narrative	Translated narrative
Discharge summary Cardiology	PACIENTE DIABÉTICA, HIPERTENSA, CARDIOPATIA ISQ. COM IMPLANTE DE STENT EM DAE EM JUL/03 INTERNOU COM QUADRO DE ANGINA INSTÁVEL. TRANSFERIDA PARA O SERV DE HEMODINÂMICA, REALIZOU CAT SENDO SUBMETIDA A ACTP EM LESÃO DE ÓSTIO DA SEGUNDA DIAGONAL. PROCEDIMENTO REALIZADO COM SUCESSO ANGIOGRÁFICO. RECEBE ALTA ASSINTOMÁTICA. Paciente ex-tabagista, vem à emergência com quadro de dispnéia progressiva, ortopnéia, dispnéia paroxística noturna, edema de membros inferiores, turgência jugular. Diagnóstico de insuficiência cardíaca, com classe funcional IV (NYHA) na chegada. Sem história de dor torácica. ECG da chegada sem alterações. Marcadores de necrose miocárdica normais. Manejado para insuficiência cardíaca com boa resposta clínica. Ecocardiograma demonstrando dilatação de cavidades (AE = 5,3 cm, DDVE = 7,0, DSVE = 5,8), disfunção sistólica (FEVE = 35%) por hipocinesia difusa, septo e parede posterior de 0,9 cm, insuficiência mitral e tricúspide leves e PSAP = 52 mmHg. Realizado investigação etiológica com sorologia negativa para Chagas, cintilografia demonstrando necrose apical, sem condições de discriminar isquemia. Optado então pela realização de cateterismo cardíaco, que revelou artéria circunflexa dominante e livre de lesões significativas; artéria coronária direita livre com sinais de aterosclerose, mas sem lesões significativas; artéria descendente anterior de pequeno calibre, com lesão de cerca de 60% no terço proximal e lesão crítica no terço médio. Após revisão do filme, observou-se tratar de lesão de difícil manejo percutâneo, devido à sua extensão e ao pequeno calibre da artéria descrita. Após discussão do caso, optou-se por manejo clínico devido ao fato do paciente não apresentar angina, ter respondido com sucesso à terapêutica instituída e não apresentar evidência clara de benefício atual com procedimento de revascularização. Impressão de que a lesão em DAE não explicaria a hipocinesia difusa apresentada pelo paciente, devendo ser portanto doença aterosclerótica coexistindo em um coração com miocardiopatia dilatada. Realizado ainda espirometria que evidenciou distúrbio obstrutivo moderado. DCE estimada em 57 ml/min. Paciente recebe alta em bom estado geral, afebril, eupnéico, em otimização do tratamento para ICC (já em uso de betabloqueador, IECA e espironolactona), com plano de ajustes de doses a nível ambulatorial. OBS: peso na alta: 76 Kg.	DIABETIC PATIENT, HYPERTENSE, ISCHEMICAL CARDIOPATHY. WITH STENT IMPLANT IN LAD IN JUL / 03 HOSPITALIZED WITH SYMPTOMS OF UNSTABLE ANGINA. TRANSFERRED TO THE SERVICE OF HEMODYNAMIC, PERFORMED CATHETERISM, SUBMITTED TO PCTA IN THE SECOND DIAGONAL INJURY. PROCEDURE PERFORMED WITH ANGIOGRAPHIC SUCCESS. ASYMPTOMATIC HOSPITAL DISCHARGE. Ex-smoker patient comes to the emergency room with progressive dyspnea, orthopnea, paroxysmal nocturnal dyspnea, lower limb edema, and jugular turgence. Heart failure diagnosis, with functional class IV (NYHA) upon arrival. No history of chest pain. ECG on arrival without change. Normal myocardial necrosis markers. Managed for heart failure with good clinical response. Echocardiogram showing cavity dilatation (LA = 5.3 cm, LVDD = 7.0, LVSD = 5.8), systolic dysfunction (LVEF = 35%) due to diffuse hypokinesia, a 0.9 cm septum and posterior wall, mild mitral and tricuspid regurgitation, and APSP = 52 mmHg. Etiological investigation with Chagas negative serology, scintigraphy showing apical necrosis, unable to discriminate ischemia. Then opted for catheterization which revealed a dominant circumflex artery free of significant lesions; free right coronary artery with signs of atherosclerosis but no significant lesions; small anterior descending artery with a lesion of about 60% in the proximal third and critical injury in the middle third. After review of the film, it was observed that it was a difficult percutaneous management injury owing to its extension and the small caliber of the described artery. After discussion of the case, we opted for clinical management because the patient did not have angina, successfully responded to the therapy instituted, and did not present clear evidence of being benefitted by the revascularization procedure. The impression that the lesion in LAD would not explain the diffuse hypokinesia presented by the patient; therefore, atherosclerotic disease coexisting in a heart with dilated cardiomyopathy. Accomplished yet spirometry that showed moderate obstructive disorder. DCE estimated at 57 ml / min. Patient is discharged in good general condition, afebrile, eupneic condition, optimizing treatment for CHF (already using beta-blocker, ACEI and spironolactone), with outpatient dose adjustment plan. OBS: weight in the high: 76 Kg
Ambulatory note Nephrology	NEFROPATIA DIABETICA EM TTO CONSERVADOR CANDIDATA A TX RENAL PREEMPTIVO LIBERADA PELA URO E ANESTESIO CANDIDATA A TX RENAL PREEMPTIVO ASSINTOMÁTICA, EXCETO PELOS SINAIS E SINTOMAS ASSOCIADOS A NEUROPATIA PERIFERICA (DIABETICA / UREMIA) SEM SINTOMAS URINARIOS AO EXAME PA 150/100 P 108 T 36 DIURESE FRR NORMAL HIPOCORADA + CPP LIVRES PC RITMO REGULAR, TAQUICARDICO ABD RHA+, PLANO, FLÁCIDO, CIC CX CST MMII PULSOS PRESENTES E SIMETRICOS	DIABETIC NEPHROPATHY IN CONSERVATIVE TREATMENT PREEMPTIVE KIDNEY TRANSPLANT CANDIDATE RELEASED BY UROLOGY AND ANESTHESIOLOGY PREEMPTIVE KIDNEY TRANSPLANT CANDIDATE ASYMPTOMATIC, EXCEPT FOR SIGNS AND SYMPTOMS ASSOCIATED WITH PERIPHERAL NEUROPATHY (DIABETIC / UREMIA) NO URINARY SYMPTOMS ON EXAMINATION BP 150/100 HR 108 T 36 DIURESE RR NORMAL PALLOR + FREE LF CS REGULAR RHYTHM, TACHYCARDIC ABDOMEN RHA +, FLAT, FLACCID, CIC CX CST LLLL PRESENT AND SYMMETRICAL PULSES
Nursing note Not defined	Pcte com RNM de crânio agendada para hoje às 23:00 h. Por volta das 21:00 h pcte apresentou quadro de confusão mental, seguida de crise convulsiva generalizada, prontamente atendido na sala de poli, com MCC + oximetria digital de pulso + PNI contínuos. Instalado O2, medicado CPM e mantido em observação no leito. Hidantalizado pela R1 Vital Brasil da neurocirurgia, procedimento realizado sem intercorrências. Pcte bastante sonolento, mantido em sala de poli e suspenso RNM por hora. Diurese espontânea, com controle através de uropen. SSVV às 05:45 h PA = 133/74mmhg, FC = 114 bpm, SpO2 = 93%. Conforme orientação da neurocirurgia, mantém observação na sala de poli sob cuidados intensivos de enfermagem. CHOQUE NAO ESPECIFICADO	Patient Skull MRI scheduled today at 23:00. At around 21:00, the patient presented with mental confusion, followed by generalized seizure, promptly treated in the multiple trauma room, with MCC + digital pulse oximetry + continuous NIBP. Installed O2, medicated as prescribed and kept under observation in bed. Hidrantalized by R1 Vital Brasil of neurosurgery, procedure performed without complications. Very sleepy patient kept in emergency room and suspended MRI for hour. Spontaneous diuresis, with uropen control. VVSS at 05:45 h BP = 133 / 74 mmhg, HR = 114 bpm, PsO2 = 93%. As directed by neurosurgery, maintains observation in the emergency room under intensive nursing care. SHOCK NOT SPECIFIED

Examples of clinical narratives included in our corpus include different types (e.g., discharge summaries, ambulatory notes, nursing notes) and medical specialties. The first column shows the original pt-br text, and in the next column, the translated version (some acronym translations may not make sense in English)

#### Document selection

The original and primary focus of the intended semantic annotation was two-fold: (i) to support the development of an NER algorithm to be used in a summarization method and (ii) to evaluate a semantic search algorithm focusing both on the cardiology and nephrology specialties. Thus, almost 500 clinical notes were randomly selected from both medical specialties (including the complete longitudinal records of two patients). To compensate for the lack of corpora for pt-br, the scope of this study was increased to support other biomedical natural language processing (bio-NLP) tasks and medical specialties. Documents from other medical areas were randomly selected to complete 1000 clinical narratives, assuming that the data were consistent and representative for the training of a ML model. Table 2 presents the number of documents per specialty. The average character token size was ~ 148 and the average sentence size was approximately ten tokens.

**Table 2:** The medical specialties frequency table

Specialty	Number
Cardiology	260
Nephrology	157
Orthopedics	126
*Not defined*	122
Surgery (general)	61
Neurology	45
Neurosurgery	32
Dermatology	23
Ophthalmology	22
Endocrinology	19
Gastroenterology	16
Otolaryngology	14
Pneumology	11
*Others*	92

The medical specialties of the selected clinical narratives were ordered according to their frequency in the corpus. Medical specialties with less than ten occurrences were grouped into “*Others*” category

Note that several documents were categorized as “Not defined” because this is one of the majority classes in the corpus received. When these documents are analyzed, it is concluded that these patients are (a) under the care of multiple medical specialties (e.g., patients with multiple traumas in the intensive care unit) or (b) in the middle of a diagnostic investigation. The specialties with less than ten documents were further grouped as “Others” (e.g., urology, oncology, gynecology, rheumatology, proctology). Regarding document types, the selected documents were represented by 126 discharge summaries, 148 admission notes, 232 nursing notes, and 506 ambulatory notes.

#### Text organization

In Table 3, available data are presented for each entry in the database (concerning first the main data source). To obtain a unique text file per entry, all the free-text fields were concatenated into a single text document to be annotated. In addition to the already-known issues in clinical texts, our database presented other problems. The medical staff were supposed to write all patient clinical notes in free-text fields. The EHR application has one textbox for each field, and these sections serve as clinical narrative sections. However, most clinicians entered all of the text in the history-of-disease field only, while leaving other fields blank, making it difficult to search for specific information in the narrative (e.g., look for family history). In addition, some text was entirely written in uppercase letters interfering directly with text processing, such as finding abbreviations and identifying proper nouns.

**Table 3:** Database entry data configuration

Field	Data type
Occurrence-id	Number
Patient-id	Number
Gender	Text
Birth date	Date
Inclusion date	Date
Discharge date	Date
Discharge type	Text
Discharge reason	Text
ICD-10	Text
Medical specialty	Text
Care reason	Text
Main complaint	Free-Text
History of disease	Free-Text
Past history	Free-Text
Family history	Free-Text
Physical examination	Free-Text
Main diagnosis hypothesis	Free-Text
Initial plan	Free-Text
Observations	Free-Text

Data fields for each EHR entry in our main data source. The fields have different data types: numerical, date, text (one-line small text), and free-text (multi-line and large text)

### Annotation schema

In this section, we describe the entire annotation schema, including the conception and evolution of the annotation guidelines, the development of a tool to support and improve the annotation workflow, and an overview of the annotation process and its experimental setup. The steps of the annotation process considered the lessons learned from other similar annotation projects reviewed in Methods section.

#### Annotation guidelines

To ensure gold standard quality, it is crucial to maintain the homogeneity of the annotation during the entire process. To provide guidance to annotators and answer possible questions, a set of guidelines were defined to explain, in detail, how to annotate each type of concept with examples to illustrate what should be annotated and what should not.

The first step was to define the information to annotate within the text. Regarding the clinical concepts, UMLS semantic types (STY) were opted for as annotation tags (e.g., “Body Location or Region,” “Clinical Attribute,” “Diagnostic Procedure,” “Disease or Syndrome,” “Finding,” “Laboratory or Test Result,” “Sign or Symptom,” and “Therapeutic or Preventive Procedure”). Table 4 presents some of the most commonly used STYs with examples.

**Table 4:** Text samples containing the most used STYs

SGR	STY	Original examples	Translated examples
Anatomy	Body Location or Region	MEIA TALA GESSADA EM MIE apresenta edema em região craniana ABDÔMEN PLANO E FLÁCIDO	Half-length plaster cast in LLL presents edema in the cranial region FLAT AND FLACID ABDOMEN
Anatomy	Body Part, Organ, or Organ Component	acesso venoso central em jugular D ACESSO VENOSO PERIFERICO EM BRAÇO DIREITO	right jugular central venous access RIGHT ARM PERIPHERAL VENOUS ACCESS
Chemicals & Drugs	Organic Chemical	Fez uso de atenolol por 3 anos cefaléia em regiao parietal bilateral que melhora com dipirona	used atenolol for 3 years headache in bilateral parietal region improved with dipyrone
Chemicals & Drugs	Pharmacologic Substance	asmatica em uso de salbutamol e budesonida	asthmatic person using salbutamol and budesonide
Concepts & Ideas	Temporal Concept	POI DE LAVAGEM + CURETA DE TECIDO NECRÓTICO Paciente em Pré-operatório de FX fêmur	WASHING IP + NECROTIC TISSUE CURETAGE Preoperative patient with femur fracture
Devices	Drug Delivery Device	cloreto de potassio a 42 ml/h em bomba de infusão	potassium chloride at 42 ml/h in infusion pump
Devices	Medical Device	AVP em MSE com soroterapia em curso SVD com diurese efetiva	PVA in LUL with ongoing serotherapy DBP with effective diuresis
Disorders	Disease or Syndrome	REFERE HIPERTENSÃO E DIABETES EM USO DE INSULINA. SINDROME DE GUILLAIN BARRE.	REFERS HYPERTENSION AND DIABETES IN INSULIN USE GUILLAIN BARRE SYNDROME
Disorders	Finding	RETORNOU DO CC LÚCIDO, ORIENTADO, COMUNICATIVO consciente, comunicativo, pupilas isocóricas fotoreagentes	RETURNED LUCID FROM SC CONSCIOUS, COMMUNICATIVE conscious, communicative, photoreagent isochoric pupils
Disorders	Injury or Poisoning	TRAUMA CRÂNIOCERVICAL APÓS QUEDA FRATURAS MULTIPLAS DA COLUNA TORACICA.	SKULL-CERVICAL TRAUMA AFTER FALL MULTIPLE FRACTURES IN THORACIC COLUMN
Disorders	Sign or Symptom	relata cefaléia SINAIS VITAIS ESTAVÉIS, REFERE ALGIA	reports headache STABLE VITAL SIGNS, REFERS PAIN
Living Beings	Patient or Disabled Group	paciente nega queixas, nega dor, dispnéia. Pcte com cultura de Secreção Tibial	patient denies complaints, denies pain, dyspnea. PTT with Tibial Secretion culture
Living Beings	Professional or Occupational Group	Orientada a equipe de enfermagem que o mesmo esta em jejum segundo a farmacêutica e o médico	Advised nursing staff that the patient is fasting according to the pharmacist and the doctor
Organizations	Health Care Related Organization	CONFORME ROTINA DA UTI RETORNOU DO CC ÀS 14:30 HRS	AS ICU ROUTINE RETURNED FROM SC AT 2:30 pm
Phenomena	Laboratory or Test Result	Glicose 335; LDH 223; Teste rápido para HIV negativo	Glucose 335; LDH 223; HIV negative rapid test
Physiology	Clinical Attribute	PA = 130/70 PESO 67,4	BP = 130/70 WEIGHT 67.4
Procedures	Diagnostic Procedure	AUSCULTA PULMONAR; MV +, RONCOS DIFUSOS EM BASES Monitorização cardíaca contínua, PAM e oximetria digital.	PULMONARY AUSCULTATION; VM +, DIFFUSED WHEEZES IN BASES Continuous cardiac monitoring, MAP, and digital oximetry.
Procedures	Health Care Activity	EM ACOMPANHAMENTO NA ENDOCRINO DO HC Internamento em janeiro por taquicardia atrial com aberrância	FOLLOW-UP ON ENDOCRINOLOGY AT HC Admission in January for aberrant atrial tachycardia
Procedures	Therapeutic or Preventive Procedure	SVD COM 100 ML DEBITO SEM GRUMOS IRC EM DIALISE	DC WITH 100 ML DEBIT WITHOUT GROUNDS CRF IN DIALYSIS
N/A	Abbreviation	CONFORME ROTINA DA UTI [Unidade de Terapia Intensiva] MEIA TALA GESSADA EM MIE [Membro inferior esquerdo]	AS ICU [Intensive Care Unit] ROUTINE Half-length plaster cast in LLL [Lower left limb]
N/A	Negation	Paciente eupnéico e afebril Paciente nega algia SEM IRRADIAÇAO	Eupneic and feverless patient Patient denies pain NO IRRADIATION

Text samples containing the most used semantic types and their corresponding semantic groups. The third column shows the original examples and the fourth column shows the translated versions. The underlined passages indicate the annotated concepts

The choice of UMLS STYs was to ensure the high-granularity of the annotation, to establish the ground-truth for the evaluation of a semantic search algorithm that labels entities using the STYs (more than a hundred types). The risk of agreement loss (as a result of the reliability and granularity trade-off discussed in Section 2) was acceptable; however, the greater coverage of the concepts in the corpus allowed the gold standard to be utilized in a higher number of bio-NLP tasks. Even when the task has low granularity, it is possible to export the actual annotations to their corresponding UMLS semantic groups (SGR). The second reason for the use of the UMLS STYs was its reliance on the UMLS Metathesaurus resource, which can serve as an important guide to annotators, as they can search for a specific concept to ensure that it is the STY they are annotating.

The UMLS REST API allows the annotation tool to automatically suggest STYs for clinical concepts. As the STYs do not cover two important bio-NLP tasks, two more types were added to our tag set, the “Negation” and “Abbreviation” tags. The first aims to identify negation cues associated with clinical concepts (already tested in the negation detection algorithm presented in a later section). The “Abbreviation” type was incorporated to help in the process of abbreviation disambiguation. It is important to emphasize that these two extra STYs can complement those adopted by the UMLS. For example, it is possible to mark the term “PA” (blood pressure) as Clinical Attribute and Abbreviation at the same time.

Sometimes, when extracting semantic meaning from clinical text, the semantic value of a concept alone is not sufficient to infer important events and situations. Hence, the annotation of the relationships is incorporated between clinical concepts and the guidelines. The relation annotation schema was partially derived from the UMLS relationship hierarchy. Unlike the concept schema, a restricted set of tags was used, instead of 54 UMLS relationship types (RTY), to simplify the relation annotation which was not our main focus. The RTYs included only the “*associated_with*” and “*negation_of*”, added to complement the Negation STY (not a UMLS RTY). There are five major non-hierarchical RTYs (i.e., *conceptually_related_to, functionally_related_to, physically_related_to, spatially_related_to, and temporally_related_to*) that connect concepts by their semantic values. They are represented using their parent RTYs only, the “*associated_with*” RTY. Depending on the selected STY, it is possible to infer the sub-types of “*associated_with*” automatically. Once the concepts and relationships were defined, an annotation script was established, whereby the annotator first labeled all of the concepts and then annotated the relations. This order was adopted because Campillos et al. [15] found that the agreement between annotators was higher when annotation was performed this way.

Deleger et al. [41] stated that the most challenging STY to annotate was “Finding,” because it is a very broad type that can correspond to signs / symptoms (e.g., “fever”), disease / disorders (e.g., “severe asthma”), laboratory or test results (e.g., “abnormal ECG”), and general observations (e.g., “in good health”). To avoid ambiguity, the definition of “Finding” was simplified. Annotators would always give preference to disease / disorders and lab result STYs over the “Finding” STYs. Only results of physical examination considered normal would be marked as “Finding” (e.g., “flat abdomen” and “present diuresis”). The abnormal ones would be labeled as “Sign or Symptom.” This can cause discrepancies between UMLS concepts and our annotation; however, it makes sense for our task. Using these definitions, the first draft of the guidelines was prepared and handed to the annotators.

**Fig. 2:**
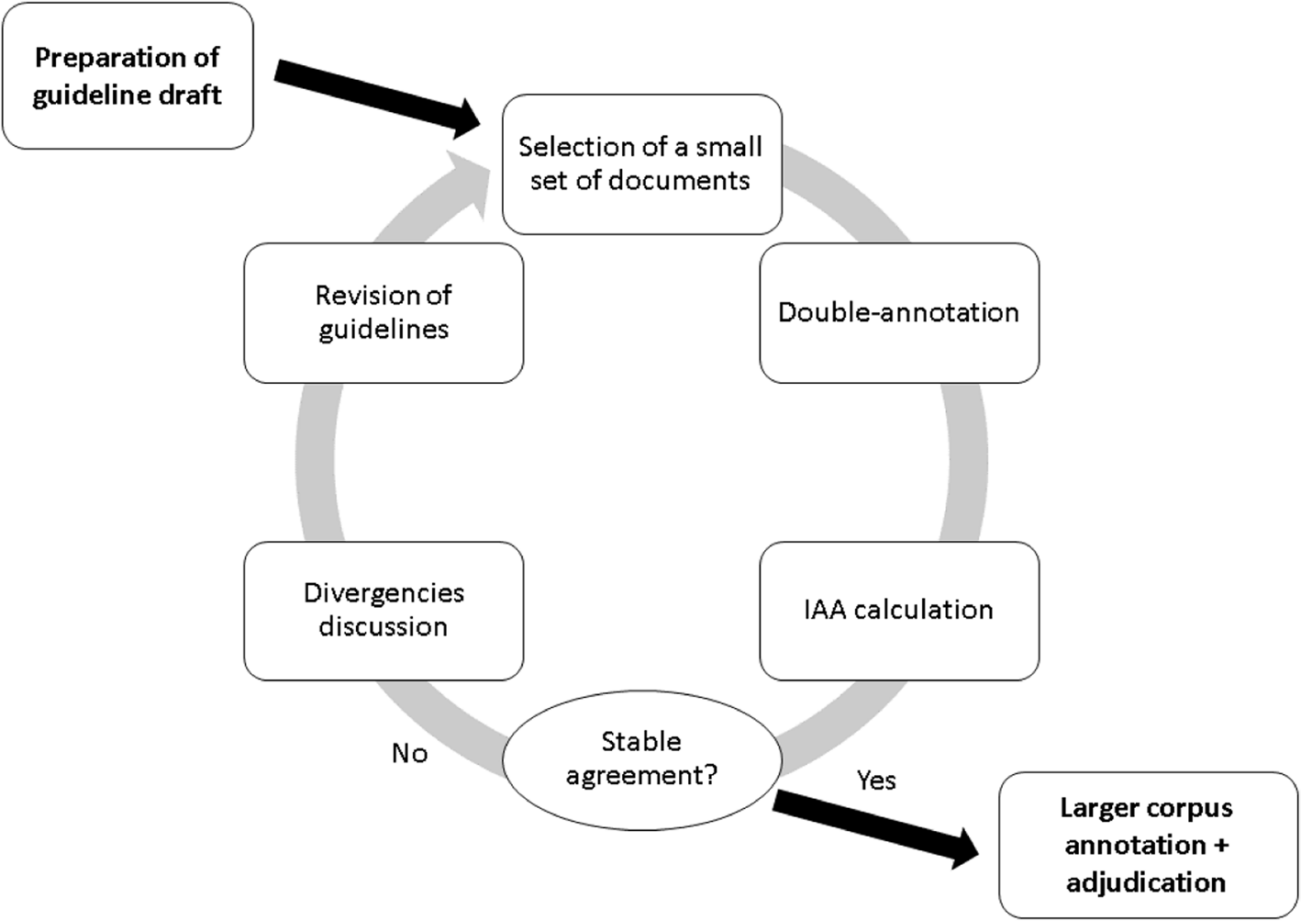
Revision and quality verification process of the annotation guidelines. The iterative process started with the first guideline draft; then, a small number of documents were double-annotated, and their inter-annotator agreement was calculated. If the agreement remained stable, then the guideline was considered good enough to proceed with the gold standard production. Otherwise, the annotation differences were discussed; the guidelines were updated; and the process was reinitiated

Furthermore, training was provided to acquaint the participants with the annotation tool and allow them to realize some of the difficulties of the process. Then, an iterative process was started to enhance the guidelines, check the consistency of annotation between annotators (more details on the inter-annotator agreement are provided in the Results section), and provide feedback on the annotators’ work. When in three consecutive rounds, the agreement remained stable (no significant reduction or improvement), it was assumed there was no room for guideline adaptation, and the final annotation process could be initiated. A flowchart of the process is shown in Fig. 2 and is similar to that of Roberts et al. [6] and others.

It is important to emphasize that even after reaching a stable agreement, the quality of the annotations continue to be evaluated and discussed among annotation teams to avoid the continuous propagation of possible errors and disparities that may arise.

#### Annotation tool

The previously discussed issues and difficulties related to clinical annotation indicate the need for an annotation tool that can ease and accelerate the annotators’ work. After analyzing Andrade et al.’s [50] review of annotation methods and tools applied to clinical texts, we decided to build our own tool. This approach ensures that all annotators can share the same annotation environment in real time and work anywhere / anytime without technical barriers (i.e., web-based applications). Furthermore, the project manager could better supervise and organize all the work and assign the remaining work to team members involved, without the need for a presential meeting because the participants had very different and irregular time schedules. Moreover, as UMLS semantic types were used in our schema, it would be desirable to use the UMLS API and other local resources (e.g., clinical dictionaries) supporting text annotation to make annotation suggestions to the user without pre-annotating it. Finally, a tool was required to fit in exactly into our annotation workflow, with the raw data input into our environment and a gold standard output at the end of the process, dispensing the use of external applications. The workflow of our tool consists of six main modules:

- Importation: import data files into the system
- Review: manually remove PHI information that the anonymization algorithm failed to catch
- Assignment: allocate text to annotators
- Annotation: allow labeling of the clinical concepts within the text with one or multiple semantic types, supported by the Annotation Assistant feature
- Adjudication: resolve double-annotation disparities in the creation of the gold standard
- Exportation: export the gold standard in JSON or XML

The annotation assistant component was developed to prevent annotators from labeling all the text from scratch by providing them with suggestions for possible annotations based on (a) previously made annotations and (b) UMLS API exact-match and minor edit-distance lookup. The UMLS 2013AA version, which was adapted to pt-br by Oliveira et al. [51] was employed. Further details on the technical aspects, module functionalities, and experiments showing how the tool affects annotation time and performance are reported in [52].

#### Annotation process

In addition to the advice and recommendations found in previous sections, similar to Roberts et al. [6], a well-known annotation methodology standard [53] was adopted. Furthermore, a guideline agreement step was added such that all the text was double-annotated with the differences resolved by a third experienced annotator (i.e., adjudicator), whereby documents with low agreement were excluded from the gold standard. Pairing annotators to perform a double annotation of a document prevents bias caused by possible mannerisms and recurrent errors of a single annotator. Moreover, it was possible to check the annotation quality by measuring the agreement between the two annotators.

It is almost impossible to achieve absolute truth in an intricate annotation effort such as this one. To reach a consistent ground truth as closely as possible, an adjudicator was responsible for resolving the differences between the annotators. It is worth mentioning that the adjudicator could remove annotations made by both annotators and did not create new annotations, to avoid hampering the creation of a gold standard based on the opinion of a single person. After the guideline maturation process, in the final development stage of the gold standard, the process was retrospectively divided into ground-truth phases 1 and 2. Annotators with different profiles and levels of expertise were recruited to provide different points of view during the guideline definition process and to determine whether there were differences in the annotation performance between annotators with different profiles.

**Fig. 3:**
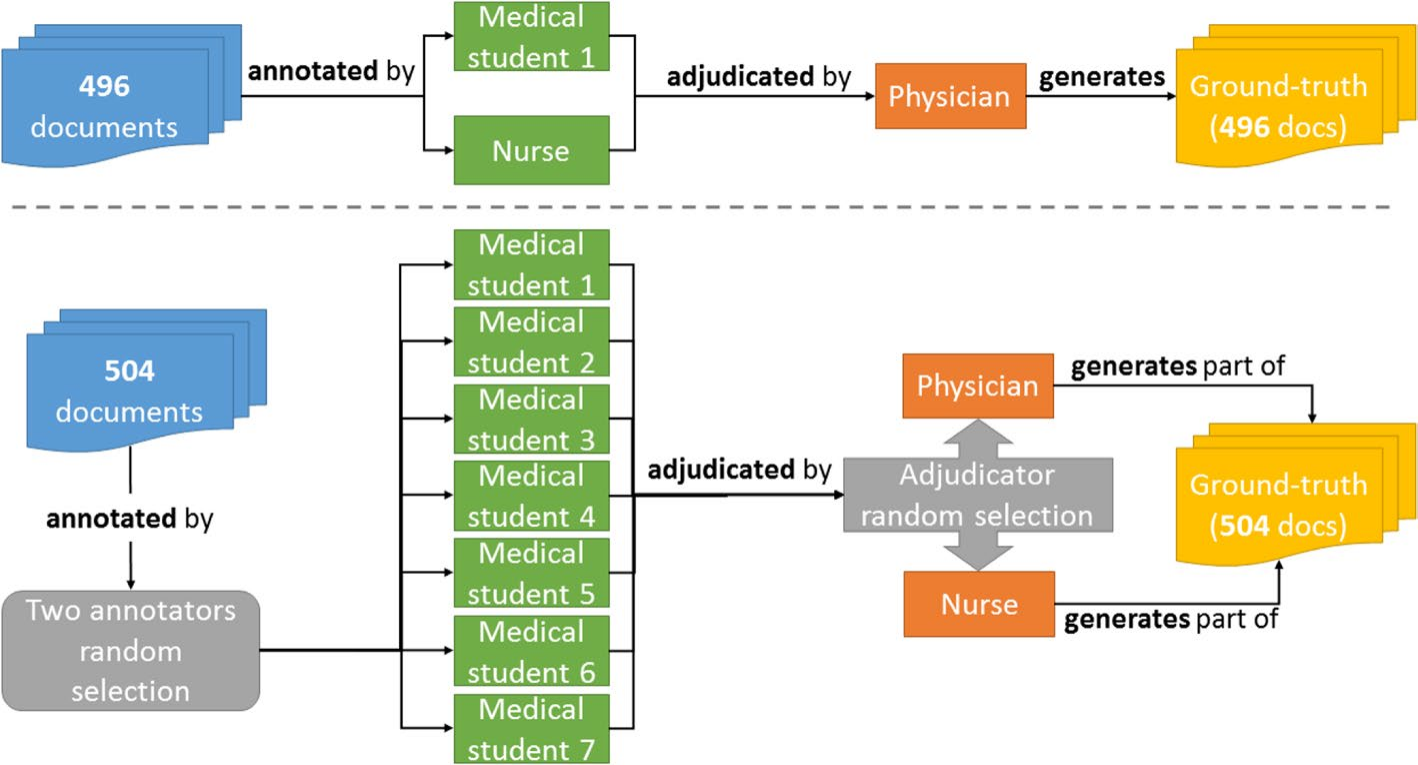
Annotation process overview. The annotation process was divided into ground-truth phases 1 and 2, which are located above and below the dashed line, respectively. The elements in green represent the annotators and orange represents the adjudicators

**Ground-truth Phase 1** included a team of three persons: (1) a physician with experience in documenting patient care and participation in a previous clinical text annotation project; (2) an experienced nurse; and (3) a medical student who already had ambulatory and EHR experience. The nurse and medical student were responsible for the double-annotation of the text, and the physician was responsible for adjudication. When the process was almost 50% complete (with 496 documents annotated and adjudicated), more people were recruited to assist in finishing the task (called **ground-truth phase 2**). An extra team of six medical students with the same background as the first one, were recruited (Fig. 3 illustrates the phases). A meeting was held to present the actual guidelines document and trained the participants on using the annotation tool.

In Phase 2, there were two adjudicators: the physician and the nurse. The nurse was added as an adjudicator, as one extra adjudicator was needed during this phase, and the nurse had more hospital experience than medical student 1. Then a homogeneous group of seven medical students was formed to annotate the texts. The physician, nurse, and medical student 1 supervised the first set of annotations for all students. The number of documents to be annotated was divided equally between the annotators and adjudicators, and the selection of double annotators for each document was made randomly, as was done for the adjudicators. It is worth noting that in addition to those mentioned, who worked directly with annotation and adjudication, there was a team of health informatics researchers who participated in supporting the annotation project with other activities, including annotation tool development, guideline discussion, and annotation feedback.

#### Corpus reliability and segmentation

Taking advantage of the fact that the entire collection of documents were double-annotated, the IAA of all the data was calculated using the *observed agreement* metric, as presented in the following equation (no need for chance-correction calculations, as described in Methods section). The following four metrics were used:

- Strict (full span and STY match)
- Lenient (partial span and STY match)
- Flexible (full span and SGR match)
- Relaxed (partial span and SGR match)

For the *strict* version of IAA, a situation was considered a match when the two annotators labeled the same textual span with equivalent semantic type. All other cases were considered nonmatches. The *lenient* version of IAA, considered partial matches, that is, annotations that overlap in the selected textual spans (with the same STY); these are counted as a half-match in the formula. The third version of IAA, called *flexible*, transformed the annotated STY to its corresponding SGR (e.g., “Sign or Symptom,” “Finding,” and “Disease or Syndrome” STYs were converted to “Disorder” SGR). prior to performing a comparison to determine whether the SGRs were equal (the textual span needed to be the same). Finally, the fourth version of IAA was *relaxed*, that is, partial textual spans (overlaps) and SGRs were considered at the same time.

$$IAA=\frac{matches}{matches+ non\_ matches}$$

To isolate the concept agreement scores from the relationship score, the relationship between IAA values was reported by considering only those relationships in which both annotators labeled two of the connected concepts. Otherwise, if an annotator did not find one of the concepts involved, the IAA relationship was directly penalized.

Boisen et al. [53] recommended that only documents with an acceptable level of agreement should be included in the gold standard, as followed in this work. However, because of the scarcity of this type of data in pt-br bio-NLP research, and because the limited amount of annotated data is often a bottleneck in ML [54], no documents were excluded from our corpus; instead the documents were segmented into two, namely gold and silver. This division was made based on the IAA values of each annotated document such that documents with an IAA greater than 0.67 belonged to the gold standard and all others to silver. The threshold of 0.67 was selected because according to Artstein and Poesio [44], it is a tolerable value. The threshold of 0.8 is thought to be rigorous, considering the complexity of our task and the number of persons involved in it. Task complexity is explained by the heterogeneity of the data obtained from multiple institutions, medical specialties, and different types of clinical narratives. The study closest to ours in data diversity is that of Patel et al. [34], with the exception that their data were obtained from a single institution. Moreover, despite the large amount of data they used, there were differences between their study and ours; for example, they used a coarse-grained annotation scheme by grouping the STYs, which made the labeling less prone to errors. Moreover, we believe that a significant portion of errors that cause disagreements come from repeated mistakes by one of a pair of annotators. Thus, the error could be easily corrected by the adjudicator, as the examples in the following sections reveal.

## Results

This section compiles the quantitative and qualitative results regarding our corpus development and discusses some of the research findings. The IAA information used to segment the corpus is detailed and the errors found during the annotation are analyzed. Finally, the results of two bio-NLP applications that have already used the current corpus for their development are introduced and presented.

### Corpus statistics

The corpus development involved seven annotators, two adjudicators, and four health informatics researchers, for a total of thirteen team members. Our corpus comprised 100 UMLS semantic types representing the entities, two extra semantic types typifying abbreviations and negations, and two relationship types defining the relations between clinical entities. The annotation process was 100% double-annotated and adjudicated, and lasted 14 months, resulting in a corpus composed of 1000 documents (148,033 tokens) with 65,129 entities and 11,263 labeled relations. In Table 5, the corpus size is presented considering the gold / silver divisions. Tables 6 and 7 list the number of annotations per STY and RTY, respectively.

**Table 5:** Corpus size considering gold and silver divisions

Segment	Documents	Entities	Relations
Gold	613	41,588	7344
Silver	387	23,541	3919
**TOTAL**	**1000**	**65,129**	**11,263**

Number of documents, entities, and relations for each corpus division (i.e., gold and silver)

**Table 6:** Number of annotations per STY

SGR	STY	Entities
Anatomy	Body Location or Region	1452
Anatomy	Body Part, Organ, or Organ Component	1373
Chemicals & Drugs	Organic Chemical	2000
Chemicals & Drugs	Pharmacologic Substance	3013
Concepts & Ideas	Quantitative Concept	3953
Concepts & Ideas	Qualitative Concept	500
Concepts & Ideas	Temporal Concept	1663
Devices	Medical Device	1617
Disorders	Disease or Syndrome	2650
Disorders	Finding	6867
Disorders	Injury or Poisoning	521
Disorders	Sign or Symptom	4707
Living Beings	Patient or Disabled Group	844
Living Beings	Professional or Occupational Group	720
Organizations	Health Care Related Organization	639
Phenomena	Laboratory or Test Result	3079
Physiology	Clinical Attribute	1128
Procedures	Diagnostic Procedure	2012
Procedures	Health Care Activity	2763
Procedures	Therapeutic or Preventive Procedure	4791
N/A	Abbreviation	12,629
N/A	Negation	2676

The number of entities annotated per semantic type and the corresponding semantic groups for the entire corpus, considering the most frequent ones

**Table 7:** Number of annotations per RTY

RTY	Relations
associated_with	9693
negation_of	1570

The number of relations per RTY for the entire corpus

### Inter annotator agreement

**Fig. 4:**
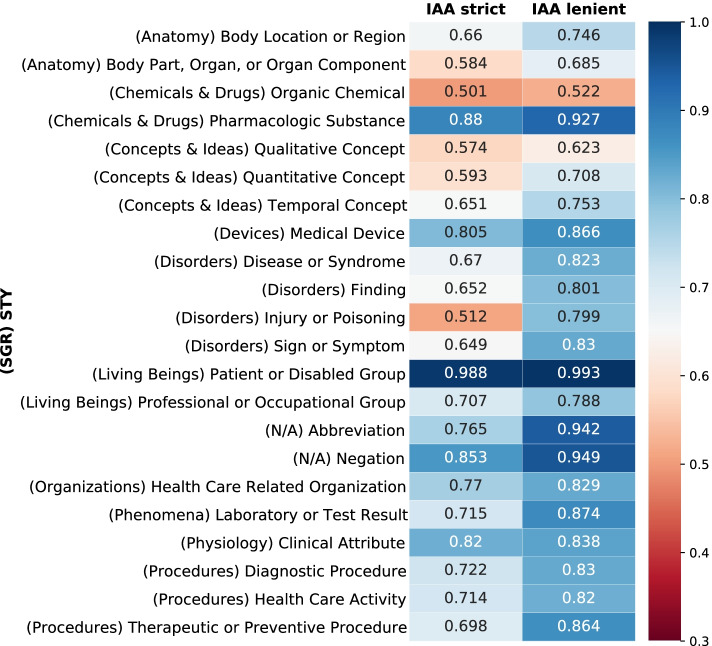
Average IAA values for the most frequent STYs. The average IAA scores for the most frequent semantic types and their corresponding semantic groups (in parentheses). The heat map indicates the highest values in blue and the lowest values in red

The average agreement between all 1000 double-annotated documents in the corpus was calculated using four different IAA versions for the concepts (strict, lenient, flexible, and relaxed) and the regular version for relations. An average IAA of ~ 0.71 for the *strict* and ~ 0.92 for the *relaxed* version were achieved in the concept annotation task. For the relations, the IAA was ~ 0.86. In Table 8, the average IAA values are detailed for the entire corpus, and Fig. 4 displays the average agreement for the most frequent STYs. Table 9 shows the IAA per RTY.

**Table 8:** Average IAA values for the entire corpus

IAA type	IAA
Strict (full span + STY match)	0.708
Lenient (partial span + STY match)	0.834
Flexible (full span + SGR match)	0.774
Relaxed (partial span + SGR match)	0.921

Average IAA values considering the four different IAA types for the entire corpus

**Table 9:** Average IAA values per RTY

RTY	IAA
associated_with	0.823
negation_of	0.914

The average IAA scores per RTY for the entire corpus

The results reveal that even with a complete annotation environment with a refined set of guidelines, use of a personalized annotation tool, clinically trained annotators, and constant reliability analysis, it is extremely difficult to reach perfect agreement. Overall, we believe the corpus built is of good quality with IAA values comparable to those of other clinical semantic annotation studies considering the specifics (described later in this section). It is worth noting that other studies evaluated their corpus by calculating the agreement between annotators and adjudicators, which typically produces superior agreement compared to IAA, as in Bethard et al. [31], who achieved 0.73 IAA and 0.83 annotator-adjudicator agreement. Another important detail is that 100% of our documents were double-annotated, not just portions of the corpus like most of the related work, which affects the final results manifested by below average agreement values during specific project phases (e.g., because of a new annotation team or guideline changes), making us believe in the trustworthiness and homogeneity of our corpus.

The IAA scores by STY (shown in Fig. 4) corroborate with other authors’ regarding the difficulty of differentiating between entity types. For example, “Disease or Syndrome” strict IAA was ~ 0.67 and “Pharmacologic Substance” was ~ 0.88, probably because the first is mainly composed of multi-word expressions, and the second is composed of single tokens. The agreement calculation used “less exact” approaches (considering SGRs over STYs) because there was a need to compare the results with those of other clinical semantic annotation studies that grouped the label categories into a few coarse-grained types, like the MERLOT, MiPACQ, and MedAlert corpora [15, 18, 36]. Our approach allows the annotator to use all the semantic types of UMLS, which are more error-prone, particularly when considering STYs in the same branch of the UMLS hierarchy tree (e.g., “Sign or Symptom” and “Finding”). It should be emphasized that in addition to the granularity issue, our corpus development faced other challenges regarding complexity. Using documents from multiple institutions raises some additional difficulties, such as dealing with different text formats specific to institutional workflows and new sets of local abbreviations or acronyms. To the best of our knowledge, no other clinical annotation study has covered documents from multiple institutions.

Handling documents from multiple medical specialties (e.g., endocrinology and dermatology) makes annotators’ training and abstraction significantly more difficult. With every new document they annotate, the chance of finding new, challenging, ambiguous exceptions is higher than documents covering topics in a single medical specialty. The CLEF corpus [6], for instance, covers only narratives from patients diagnosed with neoplasms. Document type diversity could also influence the annotation process, as the documents are produced at different times during the care workflow and are written by distinct medical professionals (physicians, nurses, medical students, or interns), which can sometimes cause interpretation problems because of the different perspectives.

Considering all these challenges and particularities of our corpus, the results were compared with previous initiatives and the IAA values of the entity and relation annotation of each corpus were compiled (see Table 10). The IAA percentage difference for entity annotation ranged from 2.8 to 18.3% using the strict match and from 3.6 to 23.2% for lenient match. Except for MiPACQ, all other corpora had better IAA values in strict match, probably because of the issues previously mentioned in this section. However, when the lenient match scores were compared, our corpus outperformed all other corpora except IxaMed-GS, which had the best results among all of them. This led us to believe that our annotators had more trouble in defining the correct text spans than the ones in other projects because with the partial span match approach, our results improved by 16.9% (from 0.71 to 0.83), whereas improvements of other corpora ranged from 3.8 to 8.6%. It is not clear whether this is as a result of a lack of proper guideline definition, annotators’ experience, or even the document types used (examples of annotation span issues are detailed in the next section).

**Table 10:** Comparison between similar clinical annotation projects

Corpus	Type	Strict	Lenient	Flexible	Relaxed
**CLEF** [6]	**entities**	0.77 (8.5%)	0.80 (−3.6%)	0.77 (0%)	0.80 (−13.0%)
	**relations**	–	0.75 (−12.7%)	–	–
**IxaMed-GS** [14]	**entities**	0.84 (18.3%)	0.90 (8.4%)	0.84 (9.1%)	0.90 (−2.2%)
	**relations**	–	0.82 (−4.6%)	–	–
**MERLOT** [15]	**entities**	0.79 (11.2%)	–	0.79 (2.6%)	–
	**relations**	–	0.78 (−9.3%)	–	–
**MedAlert** [18, 55]	**entities**	0.80 (12.6%)	–	0.80 (3.9%)	–
	**relations**	–	0.66 (−23.2%)	–	–
**MiPACQ** [36]	**entities**	0.69 (− 2.8%)	0.75 (−9.6%)	0.69 (−10.4%)	0.75 (−18.5%)
	**relations**	–	–	–	–

The percentage difference in performance between the proposed corpus and other clinical annotation projects is shown in parentheses. Note that the IAA values for Flexible and Relaxed matches are copies of Strict and Lenient scores, respectively to be able to report the percentage difference between our values and those of other authors who did not calculate these metrics specifically

With the flexible and relaxed match instead of strict and lenient for entity annotation, the results improved from 0.71 to 0.77 for flexible vs. strict, and 0.83 to 0.92 for relaxed vs. lenient. In that setting, our evaluation is fairer because the corpus granularity and complexity are more similar to other works, and thus, our results are more similar to those of other studies. Compared with the CLEF corpus, the same IAA was achieved for strict vs. flexible, while the results for lenient vs. relaxed improved by 13%. MERLOT and MedAlert displayed slightly better results (2.6 and 3.9%, respectively). IxaMed-GS still had a 9.1% advantage for strict vs. flexible, perhaps because it is the most specific and least in-depth corpus compared to the others, but even so, it has a 2.2% disadvantage for lenient vs. relaxed. Finally, our corpus surpassed MiPACQ’s results by 10.4 and 18.5% using the flexible and relaxed approaches, respectively.

For relation annotation, the scenario is entirely different from entity annotation probably because a more straightforward set of relations was used in our case compared to other corpora (except IxaMed-GS, which used only two relation categories as ours), whereby better results were obtained for relation annotation, with the percentage difference ranging from 4.6 to 23.2%.

### Error analysis

The error (or disagreement) analysis showed the most common mistakes impacting the agreement results. Error analysis was performed by the health informatics team continuously during the annotation process to provide feedback to the annotators on their work. Because performing a full error analysis for the entire corpus would be highly time-consuming, only parts of the documents in which agreement had not reached the 0.67 IAA threshold were analyzed. Moreover, the adjudicators were already aware of persistent errors. As expected, a large number of errors occurred at the beginning of the annotation phases (i.e., ground-truth phases 1 and 2) because despite the training, the annotators were still getting used to the annotation process and using the guideline document. Another common aspect of most of the disagreements is that they were not conceptual, that is, the disagreement did not originate from the semantic value given to the clinical entity, but rather from the different word span selection (term boundaries) generally associated with omission or inclusion of non-essential modifiers and verbs to a term (e.g., “*o tratamento*” vs “*tratamento*” labeled as “Therapeutic or Preventive Procedure” – “*the treatment*” vs “*treatment*”).

The high granularity of the STYs caused two types of annotation disparity. The first concerned annotations using different STYs with close semantic meanings because they are directly related to the UMLS hierarchy. One of the most recurring errors of this type was related to “Finding” and “Sign or Symptom,” even with the simplification in our guidelines stating that: annotators should always give preference to disease / disorders and to lab result STYs over “Finding” STY; the results of physical examinations considered to be normal should be marked as “Finding”; the abnormal ones should be labeled as “Sign or Symptom.” Another example of this type of error is when the annotators had to decide between “Medical Device” and “Drug Delivery Device” like with the “*infusion pump*” device. The second type of error associated with high granularity occurred because some uncommon concepts could be labeled with some infrequent STYs not remembered by the other annotator (e.g., “Element, Ion, or Isotope,” “Age Group,” “Machine Activity”).

Erroneous decomposition of multiword expressions occurred even with numerous examples explicitly described in the guidelines, especially when one annotator thought a compound term should be labeled as a single annotation, whereas the other annotator thought two or more different terms (annotations) would be more appropriate. There was no unique rule to follow in this case, as it depended on the context. Perhaps this was the reason for this type of error. For instance, the term “*Acesso venoso central direito*” (“*right central venous access*”) needs to be decomposed as “*right*” (spatial concept) and “*central venous access*” (Medical Device), but some annotators simply annotated all terms as “Medical Device.” Other terms do not need to be decomposed as “*DRC estágio V*” (“*Chronic kidney disease stage 5*”) that must be annotated as “Disease or Syndrome.”

Some errors were caused by the ambiguity of certain words that caused misinterpretation of meaning, and this occurred mainly in abbreviations. For instance, “*AC*” could be “*ausculta cardíaca*,” “*anticorpo*” or “*ácido*” – “*cardiac auscultation*,” “*antibody*” or “*acid.*” The term “*EM*” could be “*Enfarte do miocárdio*,” “*Esclerose múltipla*” or “*Estenose mitral*” – “*Myocardial infarction*,” “*Multiple sclerosis,*” or “*Mitral stenosis.*” There were also simple omission errors of certain concepts during the analysis.

In summary, STY performance (Fig. 4) reflects the complexity of each STY; for example, the “Pharmacologic Substance” is composed mainly of single-word terms, and “Patient or Disable Group” has just a few terms encompassed by it, explaining the high IAA scores, unlike “Finding” and “Sign or Symptom” that have a high frequency and very similar interpretations.

### Bio-NLP tasks application

The functionality of an annotated corpus can be tested by applying it to downstream NLP tasks. This section provides a brief overview of two bio-NLP studies that have already used the corpus presented in this work to train an ML algorithm. The main objective was to prove the consistency and usefulness of our corpus as a rich resource for pt-br clinical tasks and not to present it as a state-of-the-art algorithm.

#### Negation detection

One constant subject in bio-NLP research is negation detection, which is often a prerequisite for information extraction tasks because of its important role in biomedical text (e.g., defining the presence or absence of a disease for a patient). Dalloux et al. [56] proposed a cross-domain and cross-lingual negation and scope detection method, in which they used a supervised learning approach supported by a BiLSTM-CRF model with a pre-trained set of word embeddings. To train and assess their method within the pt-br clinical scope, they used a segment of our corpus with negation-related annotations. This includes not only the negation cue labeled with the “Negation” STY, but the concepts related to it using the relation “Negation_of” so that detecting the negation scope would be possible. They achieved an F1 score of 92.63 for negation cue detection, which is very similar to the result of the model when trained on texts from clinical trials in Portuguese, which reached an F1 score of 88.67. Regarding negation scope detection, they achieved an F1 score of 84.78 for a partial match and 83.25 for an exact match.

#### Clinical named entity recognition

One of the most important functions of bio-NLP is to identify and extract clinical entities within the text. This type of algorithm (i.e., NER) can support many methods, such as medical concept extraction, biomedical summarization algorithms, and clinical decision support systems. Souza et al. [57] described their preliminary work with promising results on exploring conditional random field (CRF) algorithms to perform NER in clinical pt-br texts. They used different fragments of our corpus and different annotation granularities (STYs and SGRs) to train and evaluate their model. Considering the best results in the exact-match approach, they achieved a 0.84 F1 score for “Pharmacologic Substance” and 0.71 for “Abbreviation” STYs, which is in line with our IAA scores. For the SGRs “Disorder” and “Procedure,” they achieved F1 scores of 0.76 and 0.70, respectively.

Schneider and colleagues [58], developed a language model for clinical NER using transfer learning (i.e., BioBERTpt). They fine-tuned a model for the NER task and the clinical domain using SemClinBr. Thus, they achieved better results than previous CRF studies: improvements of + 2.1 in accuracy, + 11.2 in recall and + 7.4 in F1.

## Discussion

Despite the extensive qualitative and quantitative analyses of the results and recognition of the reproducibility of our corpus, the study has limitations, and future work needs to be discussed. Because the guidelines were created from scratch, they went through a slow process of evolution, along with the maturation of the annotators after an extended period of annotation and analysis of new cases. To solve the inconsistencies generated by constant guideline updates throughout annotation, Campillos et al. [15] executed homogenization scripts to track and fix some of these irregularities. The corpus was maintained as the adjudicators delivered it, but with the final guidelines and corpus in hand, one can run scripts to harmonize annotations. Following discussions with the annotators, it was realized that the annotation task in clinical pt-br texts does not present any additional challenges compared with English texts, and this is reinforced by the IAA scores which are similar to those in previous studies.

Furthermore, it was concluded that the annotation tool was essential in previous studies with regard to project-time constraints. These studies claim that annotation suggestions based on previously labeled terms and UMLS API saved considerable amount of time. However, by analyzing the annotation errors, it was verified that the annotation assistant helped spread some inconsistencies throughout the corpus. This was because, at some point, the annotation assistant became a very trusted feature for the annotators, allowing it to be used quickly and carelessly by users, without the annotators checking to see if the assistant’s suggestion was really valid. In addition, the web-based annotation tool helped ease the complex logistics (already discussed in [6]) of training, monitoring, and coordinating several annotators at different locations and times.

Thus, suggestions on guideline changes to be followed by all annotators were overwhelming based on annotations made prior to the guideline update. Therefore, the use of the annotator assistant feature is beneficial; however, it should be used carefully. The UMLS API suggestion feature prevented the annotators from searching for a concept in the UMLS Metathesaurus browser, which was one of the complaints made by Deleger et al. [41] who indicated that access to an online browser slows down the annotation process. Another issue common to their study and ours was that, occasionally, the UMLS STY assignment was confusing, as the concept “chronic back pain,” is defined as a “Sign or Symptom,” but “chronic low back pain” is defined as a “Disease or Syndrome”. Additional types for “Negation” and “Abbreviation” annotation gave our corpus an even greater coverage of clinical NLP tasks, as proved in its application to a Negation and Scope detection algorithm. Moreover, as a secondary contribution, Negation cues and abbreviation dictionaries were built for further research (e.g., abbreviation expansion [59]), which will be available as the SemClinBr corpus. To replicate this annotation task and address the difficulties associated with both annotation time and guideline refinement, the number of STYs can be reduced by grouping them into SGRs, as proposed by McCray et al. [37] and applied to other annotation efforts, such as those of Albright et al. [36].

The recruitment and extensive use of medical students in annotation helped us finish the annotation considerably faster than exclusively depending on physicians and health professionals. In some sense, this reinforces Xia and Yetisgen-Yildiz’s claim [16], in which medical training is not the only factor to consider for biomedical annotation tasks. To assess the usefulness of our corpus, it should be applied to many other clinical NLP tasks, in particular, to sequence-labeling tasks, to measure the correlation between the algorithm accuracy and IAA for each semantic type. To cover temporal reasoning tasks [60], our annotation schema will be expanded to create a subset of this corpus with temporal annotation. The availability of the corpus to the scientific community will allow not only our research group, but other researchers to complement and adapt the SemClinBr annotations according to their needs, without having to start an annotation process from scratch, as Osborne et al. (2018) [61] did when normalizing the ShARe corpus, or Wagholikar et al. who used pooling techniques to reuse corpora across institutions [62]. Furthermore, the effect of the corpus homogenization process on the performance of these NLP/ML algorithms needs to be determined.

## Conclusion

In this work, the entire development process of SemClinBr, a semantically annotated corpus for pt-br clinical NLP tasks, is reported to provide a common development and evaluation resource for biomedical NLP researchers. To the best of our knowledge, this is the first clinical corpus available for pt-BR. Similar annotation projects were surveyed to identify the common steps and lessons learned to avoid mistakes and improve our work. Furthermore, the corpus has certain aspects that cannot be identified in other projects (i.e., multi-institutional texts, multiple document types, various medical specialties, high granularity annotation, and a high number of documents), which makes our annotation task one of the most complex in the clinical NLP literature, considering its generality. The data selection, design of the annotation guidelines (and its refinement process), annotation tool development, and annotation workflow are described, in detail. The reliability and usefulness of the corpus were assessed using extensive quantitative and qualitative analyses, including agreement score calculation, and error analysis, for application of the corpus to clinical NLP algorithms. Finally, in our opinion, SemClinBr, as well as the developed annotation tool, guidelines, and Negation / Abbreviation dictionaries, can serve as a background for further clinical NLP studies, especially in the Portuguese language.

## Data Availability

The resources generated in this study are available in the GitHub repository. To use the corpus, it is necessary to complete a request form with a license agreement for use only in scientific and non-commercial research, to comply with the restrictions of the ethics committee of the institution.

## Abbreviations

Bio-NLP: Biomedical natural language processing
CL: Computational linguistics
CLEF: Classical e-science framework
EHR: Electronic health records
HIPAA: Health Insurance Portability and Accountability Act
IAA: Inter-annotator agreement
MDLRM: MedAlert Discharge Letters Representation Model
MERLOT: Medical Entity and Relation LIMSI annotated Text corpus
ML: Machine Learning
NER: Named entity recognition
NLP: Natural language processing
PHI: Personal health information
pt-BR: Brazilian Portuguese
RTY: Relationship type
SGR: Semantic group
STY: Semantic type
THYME: Temporal Histories of Your Medical Events
UMLS: Unified Medical Language System
